# Use of Wavelet Transform to Detect Compensated and Decompensated Stages in the Congestive Heart Failure Patient

**DOI:** 10.3390/bios7030040

**Published:** 2017-09-20

**Authors:** Pratibha Sharma, Kimberly Newman, Carlin S. Long, A. J. Gasiewski, Frank Barnes

**Affiliations:** 1 ECEE, University of Colorado, Boulder, CO 80309, USA; kimberly.newman@colorado.edu (K.N.); Al.Gasiewski@colorado.edu (A.J.G.); frank.barnes@colorado.edu (F.B.); 2 Medicine-Cardiology, School of Medicine, University of Colorado, Denver, CO 80204; carlin.long@dhha.org

**Keywords:** acoustic signals of heart and lungs, congestive heart failure, compensated and decompensated, wavelet transform

## Abstract

This research work is aimed at improving health care, reducing cost, and the occurrence of emergency hospitalization in patients with Congestive Heart Failure (CHF) by analyzing heart and lung sounds to distinguish between the compensated and decompensated states. Compensated state defines stable state of the patient but with lack of retention of fluids in lungs, whereas decompensated state leads to unstable state of the patient with lots of fluid retention in the lungs, where the patient needs medication. Acoustic signals from the heart and the lung were analyzed using wavelet transforms to measure changes in the CHF patient’s status from the decompensated to compensated and vice versa. Measurements were taken on CHF patients diagnosed to be in compensated and decompensated states by using a digital stethoscope and electrocardiogram (ECG) in order to monitor their progress in the management of their disease. Analysis of acoustic signals of the heart due to the opening and closing of heart valves as well as the acoustic signals of the lungs due to respiration and the ECG signals are presented. Fourier, short-time Fourier, and wavelet transforms are evaluated to determine the best method to detect shifts in the status of a CHF patient. The power spectra obtained through the Fourier transform produced results that differentiate the signals from healthy people and CHF patients, while the short-time Fourier transform (STFT) technique did not provide the desired results. The most promising results were obtained by using wavelet analysis. Wavelet transforms provide better resolution, in time, for higher frequencies, and a better resolution, in frequency, for lower frequencies.

## 1. Introduction

Congestive heart failure (CHF) is the chronic medical condition which is generally characterized by the inability of the heart to pump a sufficient quantity of oxygen-rich blood to the body. This insufficiency can result in an excess fluid buildup within the body. The excess fluid pressure causes shifts into the interstitial spaces, including the lungs, which lead to a condition called pulmonary edema. The fluid buildup can also lead to pleural effusion, shortness of breath, and fatigue; however, the presence of these symptoms or lack thereof does not confirm or refute the diagnosis of heart failure due to their lack of specificity [1]. 

CHF is one of the leading global causes of death with nearly 287,000 deaths occurring each year [2], translating into an annual estimated cost of $32 billion. Costs include health care services, heart failure treatment, and employee absences from work. A home monitoring system could provide a method for early detection of the retention of fluid in the lungs and the reduced efficiency of the heart by measuring the acoustic and electrical signals as part of a regular monitoring regime. Acoustic and ECG sensor based methods that may lead to such an improvement in care are described in this article. 

Currently, congestive heart failure is diagnosed by the presence of a combination of symptoms, which include determining the presence of excess fluid in the body (peripheral edema) and lungs (pulmonary edema), along with the associative abnormalities in functioning of the heart. The methods used to assess the presence of an increase in lung water include an examination of the acoustics of the heart and lungs, whereas the excess of body fluid is determined by the appreciation of distended neck veins, and leg swelling. However, many of the symptoms caused by congestive heart failure alone do not provide enough information to make an accurate diagnosis of the condition. 

Currently, the approach used to monitor CHF patients is to collect daily weight measurements. Most patients develop symptoms around three days before hospitalization. However, this can be mitigated with proper adjustment of diuretic medication(s) [3]. Another currently used technique for CHF monitoring is to measure the electrical impedance of lung fluid between the leads of a pacemaker, or other implanted cardiac devices [4,5,6]. This signal begins to fall 11–15 days prior to the symptom onset, and is 77% sensitive in detecting hospitalization (as opposed to weight measurement, which is only 23% effective). This improved diagnostic ability was based on outcomes from the MIDHeFT (Medtronic Impedance Diagnostics in Heart Failure Trial) [5] and FAST (fast accumulations status trial) [6] trials in humans. Sensitivity improvements were realized by measuring the impedance between the left ventricular (LV) lead and the casing since this lead was closer to the lungs. Additionally, trials in canines demonstrated that ambulatory monitoring of intra-thoracic impedance is a stronger indicator of the pending onset of heart failure [7,8] than other methods, such as weight gain. Pacemakers are implanted under the skin near the collarbone to monitor the electrical signals of the heart. Signal processing based on wavelet analysis and Hilbert-Huang transformation (HHT) is done on the electrocardiogram (ECG) signals [9] that are helpful in monitoring the heart signals. Congestive Heart Signals classification was done by analyzing long record ECG and its Heart Rate Variability (HRV) signal [10]. A key limitation of most of the approaches mentioned above is that they are limited to individuals with implanted devices. It is thus impractical for most patients. The limitations mentioned above calls for the need for an external method that can be used to monitor lung fluid retention. Currently, this is done through the use of a stethoscope.

One method that may be used to detect the presence of fluid involves the use of acoustic sensors that can detect changes in sound waves produced by fluid turbulence in the heart and lungs. Sound waves produced by the opening and closing of valves in the heart will propagate differently through lungs filled with air than in condition when filled with a viscous fluid. These differences are utilized by physicians to ascertain whether a person is healthy or suffering from an abnormality in heart or lung function. Stethoscopes are used to identify these sound differences to help identify the abnormal condition. However, the current methods of diagnosis are dependent on the skills and hearing ability of the attending physician. A home-based monitoring approach that uses automated signal processing is proposed to help to reduce the variability in classification and diagnosis. 

Variability, among the individual beats and throughout the day is another marker of cardiac health. Specifically, changes in heart rate over time are indicative of a healthy individual, while the absence of such variability is associated with a greater the risk of abnormal heart function. However, such heart rate variations pose a challenge from a perspective of data normalization and comparison. Careful study and understanding of normal variations can thus provide insight for future measurements. Jos Williams, Pio Poblete, and Hubert Pipberger studied day-to-day variations in a study conducted in 1972 on 20 subjects [11]. In the study, mean and maximal day-to-day variations of durations and amplitudes of different deflections of scalar leads as well as variations of directions and magnitudes of several QRS (A part of ECG waveform that is caused due to ventricle depolarization and repolarization of atria) and T spatial vectors (A part of ECG waveform that is caused due to ventricle repolarization) were studied using computer techniques for measurement and analysis [11]. 

The major goal of this research is to determine whether acoustic monitoring methods can aid in deriving a single step systematic approach to improve our understanding and the reliability of measurements for Congestive Heart Failure. 

In an effort to better understand the natural variability in heart and lung sounds, and to establish a baseline requirement for sensitivity, initial measurements were taken on healthy subjects over several sessions. Data was subsequently collected from CHF patients over multiple sessions in both compensated and decompensated stages to compare their heart rate variability of healthy and CHF patients, as well as the changes in heart and lung signals when fluid retention has occurred. A significant variability was observed among patients; this variability is so large that variations in measurements across a broad group of people with different body shapes can exceed variations of the condition in individual patients. Multiple sessions with the same subject are thus important so that a personalized level of variability can be determined. 

Another objective of this research is to facilitate the home monitoring of CHF by the patients themselves so that the results can be transmitted to health care providers on a real-time basis, thus potentially reducing the number of hospital visits and the associated costs. This information should also help the healthcare provider to make requisite interventions on a timely basis and to be able to inform patients about their condition remotely. 

## 2. Materials and Methods

A change in lung sounds can be detected by using a stethoscope that could be a signal of the onset of pulmonary congestion/edema [12]. One methodology for examining the lung sound could be to ascertain the pattern of lung sounds, known as crackles or rales, which were derived from the re-expansion of individual pulmonary alveoli that have been filled with interstitial fluid. These could thus be a significant source of broadband noise and an effective indicator of the condition of pulmonary edema. 

Along with the above, ECG data is collected as a means of controlling where abnormalities in stethoscope measurements can be resolved to illustrate their non-correlation component with an abnormal heart rhythm. If the ECG data has consistent traits from two different sets of recordings on the same subject taken at different times, and the acoustic signals are varying, then it could be effectively assumed with a fair degree of confidence that the acoustic properties are impacted by some other parameter [13]. In this study, the ECG signals have not been used to reject any measurement that has false acoustic traits. The reason is that measuring the acoustic signals of both the heart and lung yields two data sets that are indicative of changes in the patient conditions that support each other. 

Heart sounds are in the range of 20–150 Hz and overlap breathing sounds [14]. Thus, heart sounds interfere with the measurement of sounds generated by breathing [15]. Many studies have been done with heart and lung sound separation techniques. While heart and lung sounds have independent source signals, due to multiple reflections from lung tissues the two sounds measured at the surface appear to be a convoluted mixture [16]. The heart sounds are non-stationary signals, and thus could be ascertained on the time-frequency model [17]. Holding one’s breath results in obtaining heart signals without the interference from the lung signals that are generated by breathing.

Lung sounds are created by inspiration and expiration cycles, but they may vary based on the pathological and physiological condition of the body. These sounds, when mixed with heart sounds at low frequencies, pose a critical challenge in discriminating the points of origin [18].

The current method of diagnosis is limited to the skills and experience of the attending physician(s) and even a highly trained auscultologist may not be able to ascertain important time events and features such as frequency change [19]. Much of the research to date classifies time durations of a typical heartbeat. Currently, congestive heart failure is diagnosed by the combination of signs and symptoms, which include extra fluid in the lungs and abnormal heart sounds. The methods used to find these signs are: the examination of the acoustics of the heart and lungs, as well as distended neck veins, leg swelling, and generated cardiac pulses. However, many of the signs created by congestive heart failure alone do not provide enough information to make an accurate diagnosis. This paper proposes a method to detect the differences in stages of pulmonary congestion within a CHF patient using ECG and acoustic signals.

### 2.1. Digital Stethoscope

A digital stethoscope from Thinklabs (Model DS32A, Thinklabs, Centennial, CO, USA) is used for making the acoustic measurements of both the lungs and heart. The elements of this electronic stethoscope include a transducer, amplifier, filtering, and audio input/output. The stethoscope includes ambient noise rejection. It amplifies the captured signal to ~50 times that of the signal observed with a typical unaided ear. The full spectral range of human hearing is from ~20 Hz to ~20 kHz. The nominal frequency range of sounds produced by the human heart is ~20 to ~200 Hz. Most of the energy lies below 100 Hz. The nominal frequency range of human lung sounds is ~25 to ~1500 Hz. The “bell mode” of the digital stethoscope has a filtered frequency range of 20–650 Hz. In the “diaphragm mode”, the range is from 20 to 2000 Hz. The stethoscope rejects background noise from the external environment and captures and stores the signal electronically. The stethoscope captures signals in the range from 15 Hz to 20 kHz.

**Figure 1:**
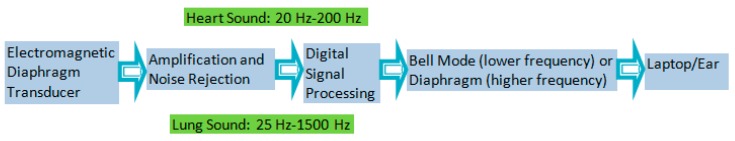
Block diagram of the DS32a digital stethoscope.

The signal to noise ratio (SNR) computed at zero gain for the two modes (bell/diaphragm) is in the range of 12 dB to 18 dB. The SNR at maximum and intermediate values of gain for the two modes (bell/diaphragm) and for the various locations of the digital stethoscope is in the range of 26 dB to 45 dB. A block diagram of a digital stethoscope is shown in Figure 1.

### 2.2. Alivecor Heart Monitoring Machine

To record the ECG of test subjects, the AliveCor heart monitoring machine was used. AliveCor is a handheld personal ECG monitoring device, which captures the electric signals emanating from the heart upon contact with the patients’ hand [20].

### 2.3. Experimental Methodology

#### 2.3.1. Study Protocol

This study focused on two groups of people. Group 1 was made up of healthy people with ages ranging from between 20 and 80 years. Group 2 was comprised of adult CHF patients. In order to obtain a 95% confidence interval using a point biserial model, a total of nine healthy subjects in group 1 and 9 CHF patients in group 2 were required. Data was collected from all subjects irrespective of gender, race, or ethnicity. The inclusion criterion for group 1 was that the subjects did not have any medical history of heart or edema related complications. The inclusion criterion for group 2 was that subjects have CHF and must have no other known heart conditions. For healthy subjects, measurements were taken at the University of Colorado at Boulder and various nearby locations. CHF patients were measured at Denver Health Medical Center. The study was approved by IRB and COMIRB. Age is a known factor in modifying heartbeat variations. Thus, an age difference amongst the healthy study group was used to help understand the expected variations for healthy heart. The subjects in both groups were informed that they could drop out of the research at any time with no penalty. It was assessed that the subject was healthy enough to be a participant in this research before the research took place.

#### 2.3.2. Procedures for Measurement

**Figure 2:**
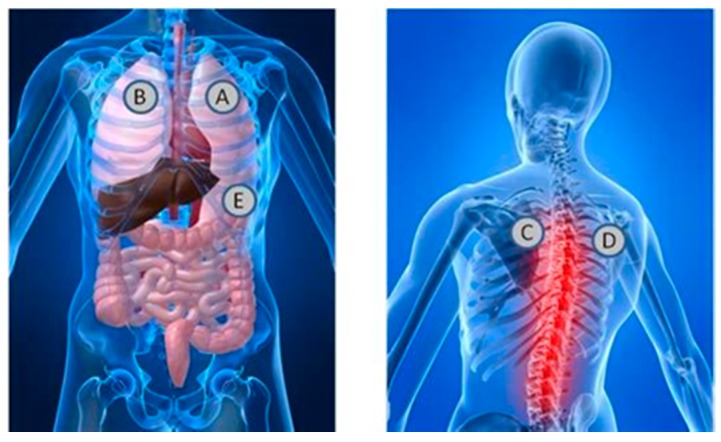
Overview of Stethoscope placement locations.

Each session required approximately 30 min for data collection. Participants were taken through the consent process and evaluated for inclusion criteria after consenting and taking a brief survey. The participants were then asked to place their hands on two electrodes of the Alivecor Heart monitoring system ECG device, while acoustic measurements were taken of the heart and lungs. A stethoscope was attached on a chest wrap with such tightness that the transducer was snug on the chest in a similar manner to a heart rate chest strap. The acoustic signals were measured at locations A, B, C, D, and E, as shown in Figure 2.

Electrical and acoustic signals were recorded and later analyzed. A minimum of two sessions per subject was desired, with no more than seven sessions in total. Data was taken from the subject in the form of cardiac electrical signals, via ECG, cardiac acoustic signals, via stethoscopes, and acoustic lung signals, via stethoscopes. The data recorded was for approximately 24 s intervals. Fourier analysis was the first signal processing tool that was performed on the data. Additionally, short time Fourier transforms and Wavelet transforms were applied to our data. 

With the ECG running, measurements were taken during breath inhalation and exhalation for acoustic lung signals with the digital stethoscope. The ECG monitor used two electrodes in direct contact with the skin. The ECG electrodes were fixed during five separate readings recorded for each session. 

#### 2.3.3. Measurement of Lung Acoustics and Heart Acoustics

The first measurement was performed with one stethoscope placed on the front left side of the chest above the pectoral muscle (location A in Figure 2), and with the subjects’ hands placed on two electrodes of the Alivecor heart monitoring machine. The stethoscope was set to diaphragm mode with gain adjusted to avoid signal saturation. Similarly, four other measurements were performed at locations B, C, D and E in the figure. The subject was asked to take deep, easy breaths for each of the measurements and hold it for approximately 24 s. Subjects were asked to inhale and exhale for four seconds during the measurement of lung signals so that the lung signals were amplified with respect to the heart signals. 

The measurement of heart acoustic signals was similar to those of lung signals except for the setting of the stethoscope and the manner in which measurements were performed. The stethoscope was set to bell mode with the gain again adjusted to avoid the saturation of the signal. Subjects (healthy people and CHF patients) were asked to hold their breath for 24 s (if possible) during the measurement of the heart so that their lung signals did not interfere with the heart signals. 

#### 2.3.4. Data Management

Recordings of the subject’s sessions were archived using only a number associated with the individual’s records. A separate notebook with each subject’s name and relation to the numbered recordings is kept in a locked filing cabinet. Electronic data is stored in a computer with password protection. Only those that are directly involved in the research and specified in the consent form as principle investigators above have access to the data. Once the study has ended, all identifiable information, including the codes, will be destroyed. The notebook with correlated code subject identification will be shredded and properly disposed of via confidential record standards. The data is reviewed and visible to only Prof. Kimberly Newman, Prof. Frank Barnes, Dr. Carlin Long, and Pratibha Sharma. There are no foreseeable outcomes that would impact the rights or welfare of the research subjects.

## 3. Signal Processing and Results

Sounds in the heart are generated by turbulence resulting from the opening and closing of the heart valves. When fluid begins to accumulate in the lungs there is a deviation in heartbeat from the nominal healthy condition, or from earlier conditions in CHF patients. As fluid in the lungs increases, the sounds are described by physicians as crackles. Heart sounds measured on the chest surface propagate through and are reflected by several media, including heart and lung tissue, fluid or air inside the lungs, bone, cartilage, and fatty tissues. The data was analyzed using Fourier transform, Short time Fourier transform and Wavelet transform.

In this study, heart sounds were measured and recorded in healthy, compensated, and decompensated CHF patients. The data from different individuals was analyzed full length signals of 24 s by plotting the power spectra. The calculated values for the discrete Fourier transform was made using a fast Fourier transform algorithm. The heart, signals were measured up to a maximum frequency of 2 kHz and then the power spectra were plotted. Visually, the plots, observed show overlapping features in the power spectra among the three groups in almost all frequency bands except 350 to 400 Hz. 

Discrimination criteria were identified by dividing the observed frequency power spectra into frequency bands, and by comparing the power at different levels with respect to a reference level, which was normalized iteratively to the maximum power for the three groups. 

**Figure 3:**
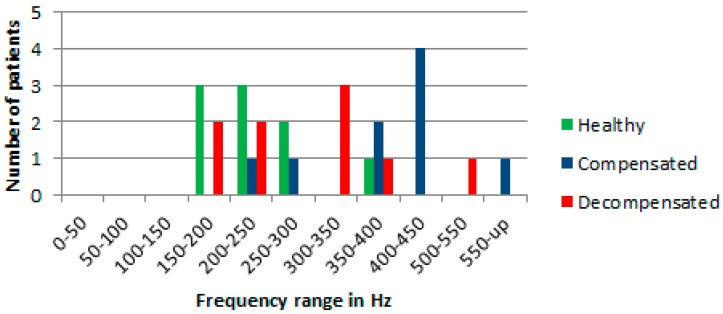
Fourier results at 2% maximum normalized power of heart signals for healthy, compensated and decompensated patients.

When comparing the power in the different frequencies, a level set at 2% of the normalized maximum power for the three groups of patients appeared to give the maximum discrimination among the three groups. A summary of the results are shown in Figure 3. 

Visually, at the level of 2% of the normalized maximum power spectra, the largest differences in the normalized power spectra between the different groups in the range for frequencies from 300 Hz to 600 Hz was observed. There is one healthy patient whose results are in the frequency band from 350 to 400 Hz, whereas the rest of the data in the range from 300 Hz to 600 Hz are for CHF patients. 

The 2% criterion identifies signals in the band of frequencies from 100 Hz to 800 Hz that seems to follow the results of Yongyudh’s paper [16]. At that level the data from healthy patients are largely between 100 Hz and 300 Hz, and CHF patients’ data are at higher frequencies. The results show that Fourier transform was successful in predicting approximately 55% for decompensated condition and around 75% for compensated condition in patients. In the measured data, there were two exceptions where healthy and CHF patients overlap. However, variability in the patients’ physiques, eating habits, heart variability, lung capacity, body fat, etc. affect the consistency of these results. For example, a small person with small lungs would be expected to have higher resonated frequencies than a large person with big lungs. Thus, in this study, the focus of the analysis was on the same CHF patients, were multiple measurements that had been made on different days.

Using a wavelet transform (WT) better time resolution is achieved for higher frequencies and better frequency resolution for lower frequencies than the Fourier transform method. The Fourier transform gives good resolution in frequency, but doesn’t give any information about the temporal spread, whereas the WT gives better temporal spread with the frequency content. The WT permits high frequency spectral features to be located in the time domain. To this end, high frequency features using WT can be better located (with less relative error) than low frequency features.

**Figure 4:**
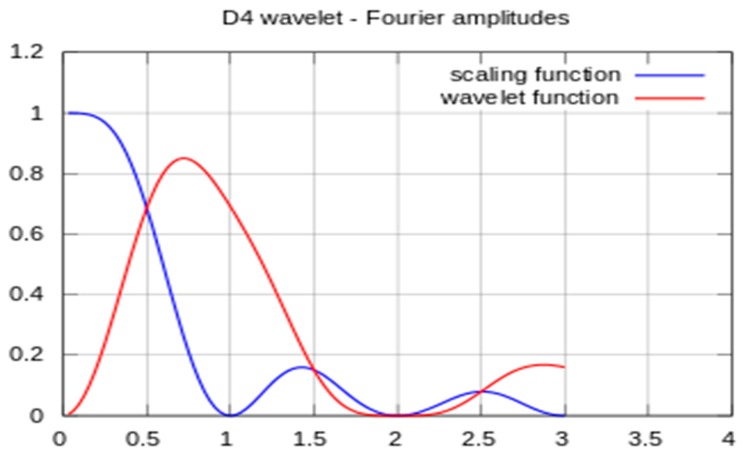
Amplitudes of the frequency spectra of the Daubechies wavelet and scaling functions.

The specific WT selected may be from any of hundreds of basis wavelets. The analysis in this study began using the Haar wavelet, which has infinite support for a given number of vanishing moments. It is well localized in time but provides poor frequency resolution. In contrast, the Shannon (or sinc) wavelet is perfectly localized in frequency but poorly localized in time. After evaluating several WT implementation approaches, the Daubechies wavelet was found to best distinguish between compensated and decompensated states, and it was adopted for this study. This method has a minimum filter length in the spatial domain for a given number of vanishing moments. The Daubechies scaling and wavelet functions (shown in Figure 4) act as low- and high-pass filter coefficients, respectively. Scaling and wavelet functions are convolved with the signal and down sampled by a factor of 2 to achieve the first level of decomposed wavelet coefficients. The low pass filter coefficients are [0.4829629131 0.8365163037 0.2241438680 −0.1294095226]. The high pass filter coefficients are [−0.1294095226 −0.2241438680 0.8365163037 −0.4829629131]. 

**Figure 5:**
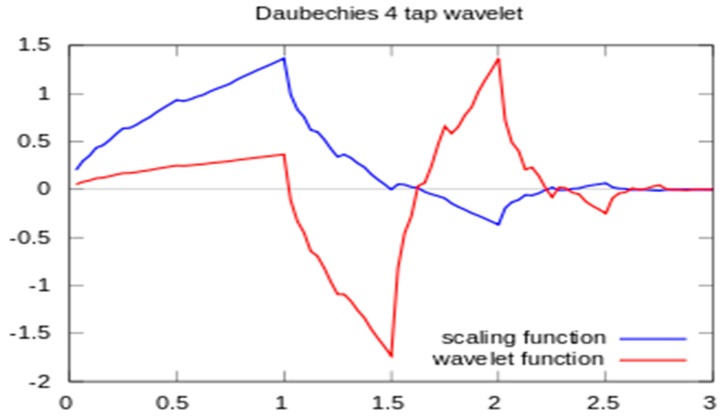
Scaling and wavelet functions.

The four decomposed details using scaling and wavelet function shown in Figure 5 are obtained after applying Daubechies wavelet analysis on acoustic heart signals. 

**Figure 6:**
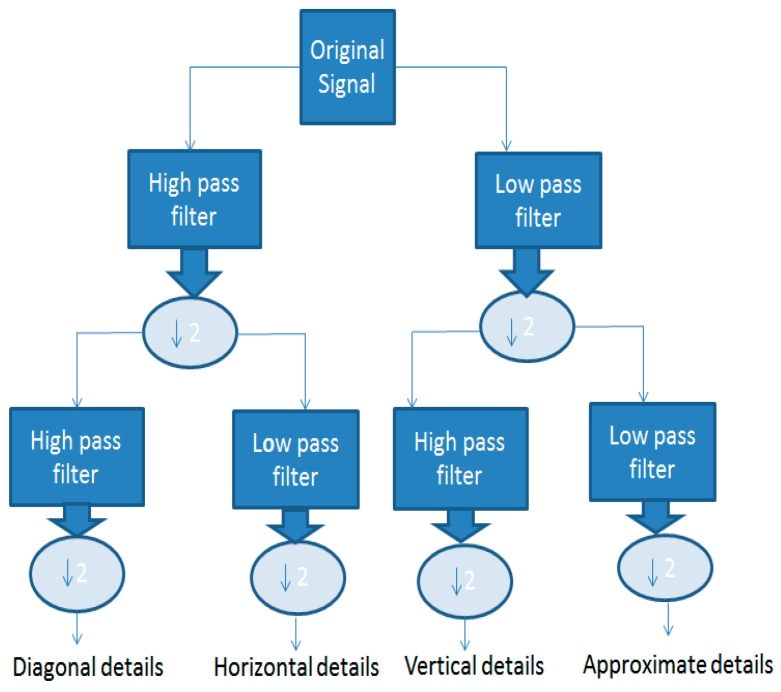
Decomposed details after applying wavelet functions.

The four decomposed details are shown in Figure 6. The first one is an approximate detail obtained by convolving our signal with a low pass filter, down sample by 2 and then convolving the result with a low pass filter and again down sampled by 2. The second one is a diagonal detail obtained by convolving our signal with a high pass filter, down sample by 2 and then convolving the result with high pass filter, and again down sampled by 2. The third is a vertical detail obtained by convolving our signal with a low pass filter, down sample by 2, and then convolving the result with high pass filter, and again down sampled by 2. The fourth is horizontal detail obtained by convolving our signal with a high pass filter, down sample by 2 and then convolving the result with low pass filter and again down sampled by 2. 

**Figure 7:**
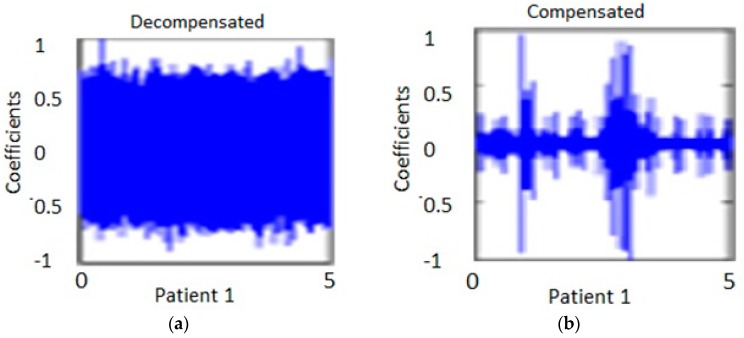
(**a**) Diagonal details (coefficients) of heart signals measured at location E for one of the patient, with decompensated state, as analyzed using the wavelet transform. (**b**) Diagonal details (coefficients) of heart signals measured at location E for the same patient as in (**a**), with measurement taken at compensated state, as analyzed using the wavelet transform.

The detailed wavelet transforms for several decompensated, marginally decompensated, compensated, and healthy subjects were analyzed. Figure 7 shows the diagonal coefficients for one patient. The measurement was taken at two different times. One was taken when the patient was in the decompensated stage and the other, at the compensated stage. The analysis was performed for 10 s duration heart signals at location E, in order to capture approximately 10 to 12 heart beats. The analysis was performed on the same CHF patients. Figure 7a shows the measurement of one individual at one period of time. Figure 7b shows the same individual from Figure 7a at a later time after the patient had been treated. 

It is to be noted that the WT details permit discrimination among the compensated and decompensated stages. Similarly, the diagonal and horizontal details show more consistent changes in the pattern of decompensated and compensated patients. The coefficents in the decompenated state are broader and are more condensed as compared to the coefficients in the compensated state for the same patient in the horizontal and diagonal representations of the data. The data show large variation in horizontal and vertical details for the decompensated patients group when compared to those of the compensated circumstances. There were two patients whose condition (decompensated or compensated) were the same for both measurements. Specifically, one patient was compensated during both measurements and another patient was decompensated for both. Excluding these two patients, the results seem to be consistency in that the spectral pattern is maintained, with the exception of one patient who showed a reverse trend. The results obtained from wavelet transform predicted with a 85% success rate in a patient’s condition from compenstated to decompensated states and vice versa.

**Figure 8:**
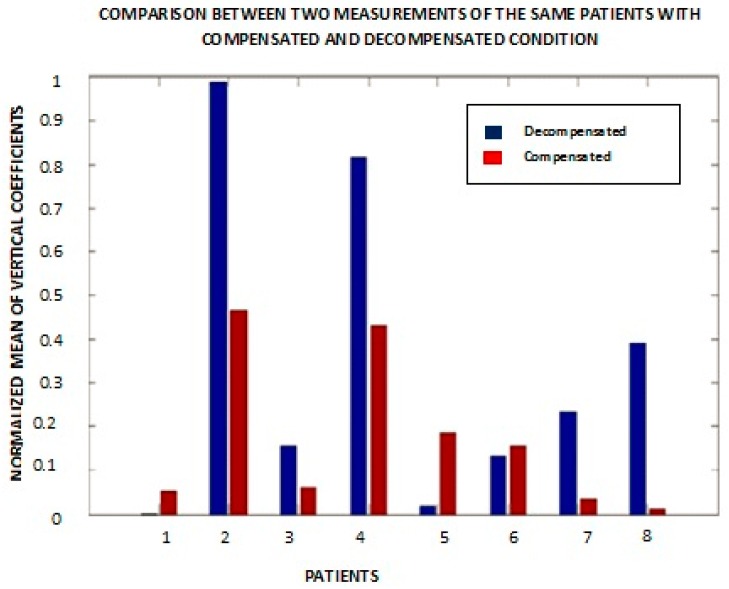
Comparative results of short duration heart signals for the same patient for the decompensated state (blue bars) and the compensated state (red bars) for patients numbered 1 to 8.

Based upon the resulting patterns, a quantitative metric to distinguish changes in the condition of individual patients was saught. The absolute value of the mean of vertical coefficients for a patient for two different measurements of the first heart sound was calculated, lPeak, S1 is 1st sound i.e., caused by turbulence that results from the closure of mitral and tricuspid valves at the start of systole. (Figure 8). Patients #5 and #7 had the same CHF condition during these measurements. Out of six patients (excluding patients #5 and #7), patients showed a lower decompensated value of the above metric compared to the compensated value. Patient #6 showed almost the same values in both cases, and patient #1 is an outlier. Medically compensated means that their symptoms and exam reflect that their CHF status is stable and essentially under control, and decompensated means they are showing symptoms and signs on the exam that they are not under adequate medical control. We note that there could be some variability in clinical classification. For example, a patient may not have fluid in the lungs and is thus is taken as a compensated case but could have accumulation of fluid in other parts of the body that are not observed with (e.g.,) a chest X-ray. Similar results were obtained from wavelet anaysis applied on lung signals. 

Figure 8 was plotted with a small duration signal from one S1 to the next S1 for eight different individuals. It shows the normalized mean of vertical coefficients with respect to compensated and decompensated patients. Patients #1 and #6 were exceptions in the sense that the magnitude of the mean of the vertical details for the compensated wavelet coefficients was greater than in the decompensated state. Patient #5 is the one with both bars showing the compensated condition.

**Figure 9:**
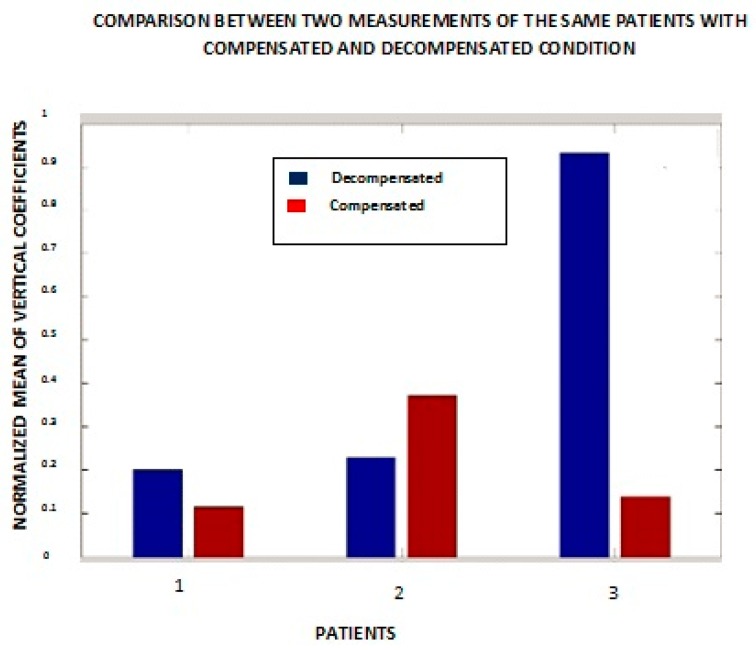
Comparative results of averaging many short duration heart signals (from one S1 sound to the next).

These experiments yield more consistent results when, instead of one heart sound, an average of multiple heart sounds are analyzed (Figure 9). The multiple sounds cover a number *n* of intervals from one S1 to the next S1. The numbers *n* = 1, 2, and 3 in Figure 9 represent patients #1, #6, and #7 from Figure 8.

The other patients are not included in this figure because these three patients are representative of the sample as a whole. It is evident from the bar graph that patient #1’s result is more consistent with our expectation that the compensated state should show a lesser magnitude of the mean of the vertical details. Patient #7 yielded similar results to those obtained from a single heart sound. Patient #6 is an outlier in both cases.

### 3.1. Correlation with Weight

Weight data for the eight patients discussed above is provided in Table 1. Patients in the compensated state are expected to weigh less than those in the decompensated state due to the loss of interstitial fluid. Patient #4, who was an outlier in the horizontal and diagonal cofficients of heart and lung signals, is consistent with the expected weight change measurements but showed opposite results with respect to the medical terms compensated and decompensated. Patient #6 also showed more coefficients in the compensated stage as compared to the decompensated stage, and was consistent with the weight change measurement, however showed opposite results with respect to the medical terms “compensated” and “decompensated”. There are 2 patients in the weight list which had implanted cardiac devices (ICD).

### 3.2. Heart Rate Variability Results

**Figure 10:**
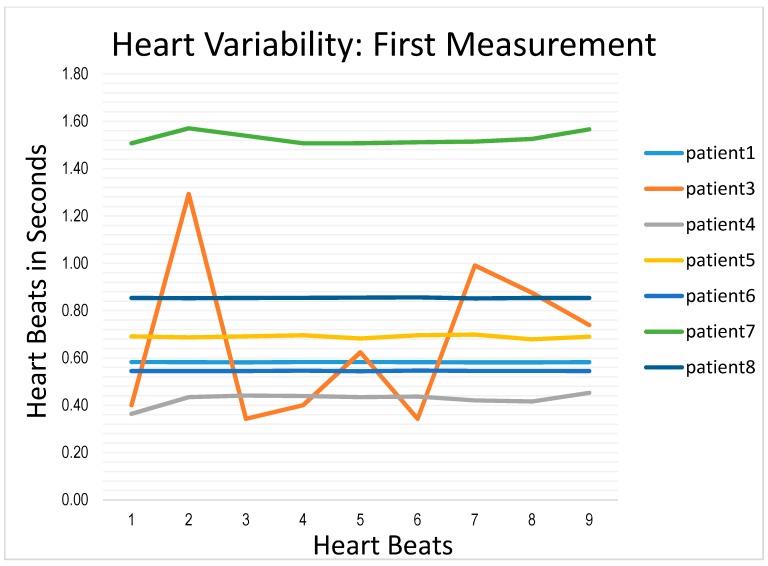
Heart Rate Variability results during the first measurement.

**Figure 11:**
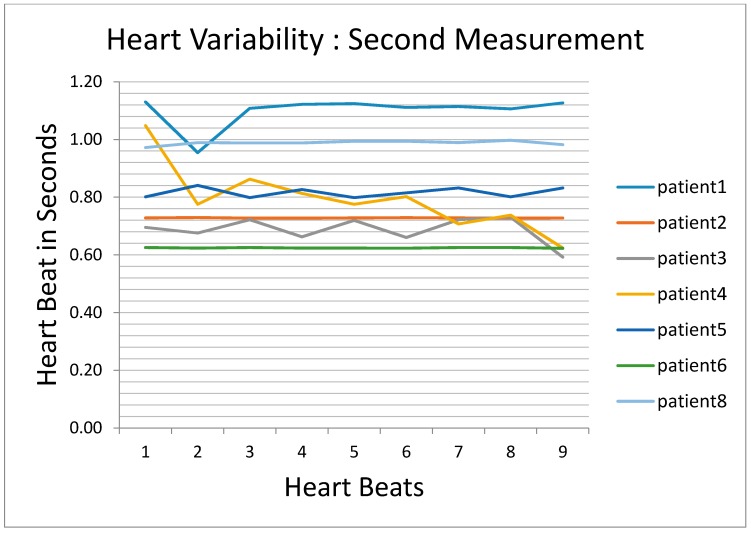
Heart rate variability results during the second measurement.

ECG signals from eight patients were collected and analyzed. A summary of these signals are shown in Figure 10 and Figure 11 for two measurements taken for the same eight patients at two different times. The ordinate in the graph has nine different beats (PQRST curve). The record for patient #2 is missing in the first graph due to equipment failure, as is the record for patient #7 in the second graph. Figure 10 shows the results for eight patients measured the first time and Figure 11 shows the results from the same eight patients measured on a second, independent occasion. Figure 10 shows the results of decompensated patients and Figure 11 shows the results of compensated patients. The curves in Figure 10 show stable heart rates as compared to the compensated patients’ heart rates in Figure 11 with the exception of patient #3. These heart rate variability results provided the similar inference as obtained by wavelet transform or weigh measurement. Heart rate variability goes up for compensated patients and goes down for decompensated patients.

## 4. Conclusions

The results of this study show that a combination of non invasive stethoscope measurements and ECG signals can be used to provide information to the doctor that reflect changes in a patient’s condition from compenstated to decompensated states and vice versa. These data are correlated with changes in weight for the majority of the CHF patients. Wavelet analysis gives promising results by showing consistent differences in spectra within the same individual. Wavelet decomposed coefficients narrowed in spectra for the same duration of signals in compensated stage as compared with decompensated stage for any given individual. Mean values of vertical coefficients taken for 1 heart beat cycle (from one S1 sound to the next S1) or over the average of multiple heart beats also indicates the differences in compensated and decompensated conditions for the same patient. Wavelet analysis appears to be a better approach than Fourier analysis to analyze the acoustic signals of the heart and the lungs. It is to be noted that the collected results showed that one of the patients was not improving during two measurements, and this was inconsistent with the medical results. However, this patient subsequently expired. The acoustic analysis, to date, does not give a clear distinction between groups of patients due to a large variabilty between mutiple patients, but it appears to be a useful tool for determining the status of a given individual patient over time.

Experiments were performed to capture the acoustic and electrical signals from healthy subjects and CHF patients by using noninvasive devices. The results can be used as a basis for analyzing a larger data set to verify the potential value of this approach in providing doctors with a way to monitor changes in a patient’s condition remotely. 

Additionally, the analysis technique to differentiate the signals between compensated, decompensated, and healthy people leads to the potential for the development of a wearable system for diagnosis of fluid retention. Finally, this research result opens the door to further research in body sensor networks, point of care devices, and provides an opportunity to create an interface for home tele-health and improved quality of care [21].

## Figures and Tables

**Table 1:** Weight measurement at two different times.

Number	Status	Weight-1	Status	Weight-2	ICD
Patient 1	Decompensated	167	Compensated	159	Yes
Patient 2	Decompensated	240	Compensated	207	No
Patient 3	Decompensated	212	Compensated	241	No
Patient 4	Decompensated	267	Compensated	267	Yes
Patient 5	Compensated	212	Compensated	213	No
Patient 6	Decompensated	210	Compensated	230	No
Patient 7	Decompensated	166	Mildly Decompensated	179	No
Patient 8	Decompensated	190	Probably Compensated	184	No
